# Activation of recombinational repair in Ewing sarcoma cells carrying *EWS*-*FLI1* fusion gene by chromosome translocation

**DOI:** 10.1038/s41598-022-19164-x

**Published:** 2022-08-30

**Authors:** Kazuhiro Tanaka, Keiji Suzuki, Kaname Miyashita, Kentaro Wakasa, Masanori Kawano, Yoshimichi Nakatsu, Hiroshi Tsumura, Mitsuaki A. Yoshida, Shinya Oda

**Affiliations:** 1 Department of Orthopaedic Surgery, Oita University, Yufu, 879-5593 Japan; 2 Department of Radiation Medical Sciences, Atomic Bomb Disease Institute, Nagasaki University, Nagasaki, 852-8523 Japan; 3 Clinical Research Institute, Cancer Genetics Laboratory, National Hospital Organization Kyushu Cancer Center, Fukuoka, 811-1395 Japan; 4 Department of Medical Biophysics and Radiation Biology, Faculty of Medical Sciences, Kyushu University, Fukuoka, 812-8582 Japan; 5 Department of Radiation Biology, Institute of Radiation Emergency Medicine, Hirosaki University, Aomori, 036-8560 Japan

**Keywords:** Genetics, Cancer genetics

## Abstract

Chromosome translocation (TL) is an important mode of genomic changes underlying human tumorigenesis, the detailed mechanisms of which are, however, still not well understood. The two major modalities of DNA double strand break repair, i.e. homologous recombination (HR) and non-homologous end-joining (NHEJ), have been hypothesized. In a typical TL^+^ human neoplasm, Ewing sarcoma, which is frequently associated with t(11;22) TL encoding the *EWS*-*FLI1* fusion gene, NHEJ has been regarded as a model to explain the disease-specific TL. Using comprehensive microarray approaches, we observed that expression of the HR genes, particularly of *RAD51*, is upregulated in TL^+^ Ewing sarcoma cell lines, WE-68 and SK-N-MC, as in the other TL^+^ tumor cell lines and one defective in DNA mismatch repair (MMR). The upregulated RAD51 expression indeed lead to frequent focus formation, which may suggest an activation of the HR pathway in these cells. Furthermore, sister chromatid exchange was frequently observed in the TL^+^ and MMR-defective cells. Intriguingly, ionizing irradiation revealed that the decrease of 53BP1 foci was significantly retarded in the Ewing sarcoma cell lines, suggesting that the NHEJ pathway may be less active in the cells. These observations may support an HR involvement, at least in part, to explain TL in Ewing sarcoma.

## Introduction

Chromosome translocation (TL) is a unique mode of chromosome aberrations underlying tumorigenesis in human cells^1^. Reciprocal and balanced TL between two distant genetic loci yields two novel and chimeric contexts, either of which sometimes encodes a ‘fusion gene’. Fusion gene-associated TL was first described in chronic myeloid leukemia (CML)^2,3^, in which a specific TL, i.e. t(9;22), is frequently observed, and *BCR*-*ABL* fusion gene transcripts are expressed in leukemia cells. One remarkable trait of the CML genome is that there are no other chromosome aberrations than the t(9;22) TL at the initial step of tumorigenesis^4^. The oncogenic transforming activities are solely attributed to the BCR-ABL chimeric protein encoded by the derivative chromosome 22, designated as ‘Ph1’^5^. TL is widely observed in human neoplasms including not only leukemias or sarcomas but also a minor population of carcinomas of the epidermal origins^6^. Molecular mechanisms underlying TL have long been discussed. However, mechanisms connecting two distant DNA double strand breaks (DSBs), i.e. ‘breakpoints’, are still enigmatic and not fully understood. Recombinational models have initially been discussed. Eukaryotic DNA recombination is regarded as comprising (a) homologous, (b) site-specific and (c) illegitimate recombination pathways. Homologous recombination (HR) is indeed one of the major modes of DSB repair in eukaryotic cells^7^. The former two recombinational modalities require sequence specificity and, in HR, sequence homology is prerequisite. Indeed, several recombinogenic sequences such as topoisomerase I/II^8–10^, chi^11^, Alu^12–15^, alternating purine/pyrimidine^16^ sequences have been found in the vicinity of the breakpoints, the structures of which are, however, complex in many cases and sometimes suggests the involvement of illegitimate recombination^17^. In case of CML, Alu-like and chi sequences encompass the breakpoints in the both *ABL* and *BCR* genes^18^. Thus, the recombinogenic sequences are distributed in the TL-involved genetic loci.

The second problem is the proximity between two genetic loci. The physical and geometrical distance between the two breakpoints in the nuclei may affect the repairability of two DSBs, either by HR or by another mode of DSB repair, i.e. non-homologous end-joining (NHEJ). From this point of view, a series of cytogenetic approaches has already been made using interphase fluorescent in situ hybridization (FISH)^19^. Kozubek and colleagues have pointed out that the *ABL* and *BCR* genes are closely distributed in the interphase nuclei of bone marrow cells^20^. TL-involved genetic loci may not be distantly arranged in the nuclei of the precursor cells. Thirdly, another noticeable problem is the repairability of DSBs. The balance between the two major modes of DSB repair, i.e. HR and NHEJ, has been discussed and is now regarded as chiefly depending on cell cycle phase, chromatin topology and subnuclear localization^7^. In addition, several components comprising the HR and NHEJ systems are known to be often affected in human cancer cells or in inherited predispositions to cancer, which are typically exemplified by *BRCA1*/*2*-associated breast and ovarian cancer (*alias* hereditary breast and ovarian cancer syndrome, HBOC)^21,22^. The balance between HR and NHEJ may also be unphysiologically biased in tumor cells or their precursor cells. Thus, molecular mechanisms underlying TL appear complex and may depend on gene, chromosome, cell-lineage and disease.

Ewing sarcoma is a class of neoplasms of the neuroectodermal origins and frequently associated with a specific t(11;22) TL encoding the *EWS*-*FLI1* fusion gene in the derivative chromosome 22. Similarly to the CML case, t(11;22) TL has been studied from various angles. Although the recombinogenic sequences such as Alu and topo II cleavage sites have been found encompassing the breakpoints in the derivative chromosome 11 and 22^23^, NHEJ is now mainly considered as a mechanism explaining t(11;22) TL, particularly after Brunet E and colleagues have shown that the *EWS*-*FLI1* fusion gene can artificially be reconstructed via the canonical NHEJ (c-NHEJ) pathway by concurrently introducing two DSBs into cells^24^. However, reciprocal and balanced TL more theoretically suggests HR mechanisms, because the HR model does require neither multicentric DSBs nor selective end-joining and the dynamic TL outcomes are simply explicable by the pivotal resolution of Holliday structures (See “Discussion” section). TL mechanisms have thus far been approached chiefly from the viewpoints of DNA, i.e. sequence, chromatin or chromosome. Another important insight for TL mechanisms is the involvement of the *trans*-acting factors, i.e. functional components of the DSB repair pathways, which, however, has not been systematically approached to date. Using cell lines established from TL^+^ Ewing sarcomas and other soft tissue and bone neoplasms, we comprehensively observed the expression of the HR and NHEJ subcomponents. The results of our observations clearly indicate activation of the HR pathway in TL^+^ Ewing sarcoma cells and may suggest the connection of the pathway to the mechanisms leading to TL and, consequently, to tumorigenesis.

## Results

### RAD51 and HR genes are upregulated in translocation-positive human tumor cells

**Figure 1:**
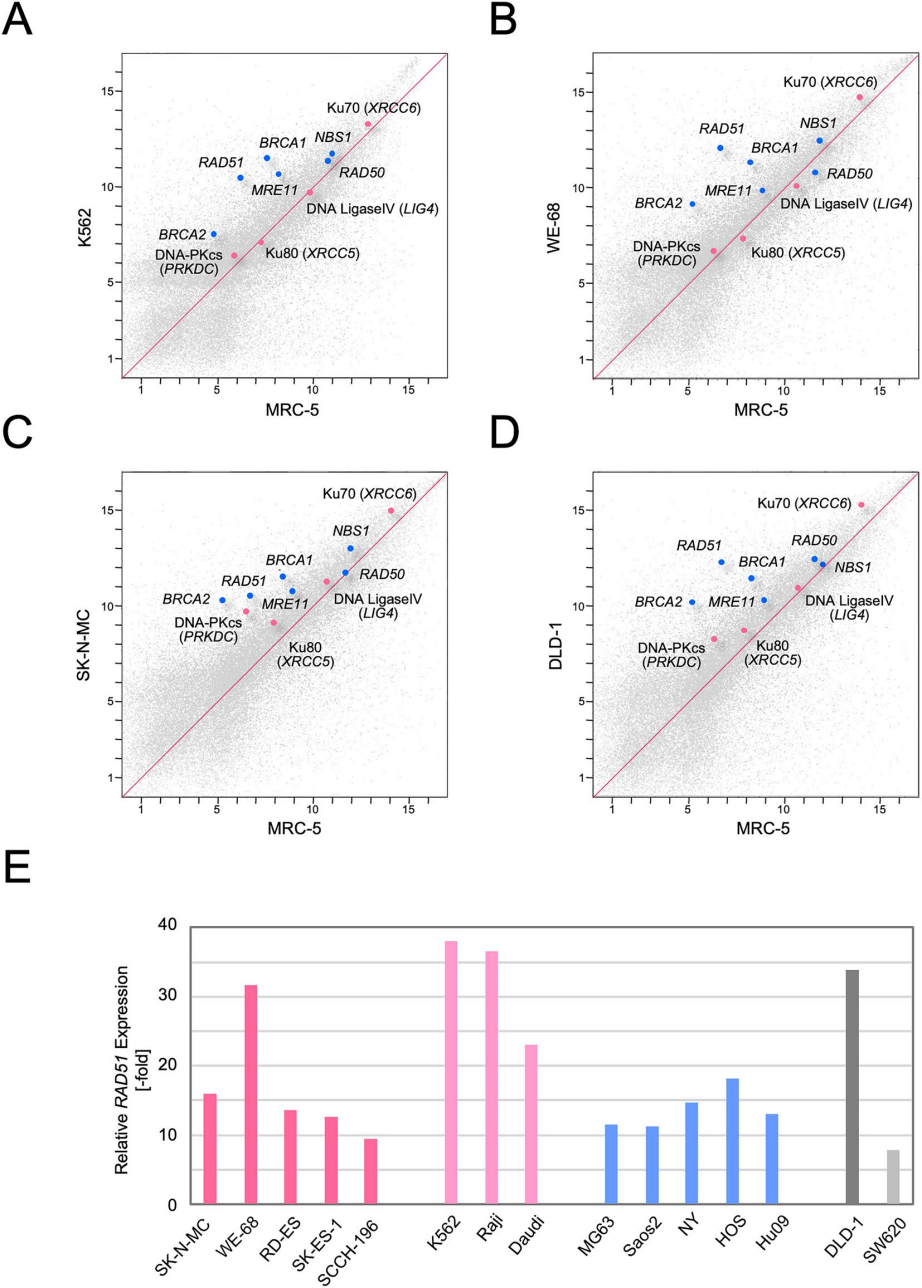
Expression microarray analyses of TL^+^ human tumor cell lines. Using an expression microarray, mRNA expression was profiled in the TL^+^ leukemia cell line, K562 (**A**), the two TL^+^ Ewing sarcoma cell lines, WE-68 (**B**) and SK-N-MC (**C**), and DNA mismatch repair (MMR)-defective colorectal cancer cell line, DLD-1 (**D**). Expression levels of mRNAs were compared with those in a primary culture of normal human fibroblasts, MRC-5, and the results were shown in scatter plots. The expression levels of the *RAD51* gene were extracted and compared among various human tumor cell lines (**E**).

In order to broadly explore the expression levels of the genes constituting the DSB repair pathways, we employed expression microarray approaches. Firstly, a leukemia cell line, K562^25^, which has been established from a Ph1^+^ CML patient and carries the classical *BCR*-*ABL* fusion gene, was analyzed. Using microarrays, expression levels of mRNAs were assessed in K562 cells, and the results were compared with those in a primary culture of normal human fibroblasts, MRC-5, a scatter plot of which is shown in Fig. 1A. Relative expression of the genes functioning in the HR pathway was remarkably and generally high in K562 cells. In particular, the expression level of *RAD51* was more than 30-times higher than in MRC-5, whereas those of *RAD50*, *NBS1* and *MRE11* were relatively low. On the other hand, expression of NHEJ genes in K562 cells was largely at the same levels as those in the control, MRC-5. Secondly, we extended our observation to the two cell lines established from Ewing sarcomas with the *EWS*-*FLI1* fusion gene, WE-68^26^ and SK-N-MC^27^ (Fig. 1B, C). Intriguingly, similar tendencies were observed in these cells. Expression of the *RAD51* and other HR genes was remarkably upregulated, whereas NHEJ gene expression was not significantly changed. Thirdly, we also analyzed another cell line established from human colorectal cancer, DLD-1, which is known as a cell line defective in DNA mismatch repair (MMR)^28^ (Fig. 1D). It is also known that HR is activated in cells defective in MMR since MMR acts as a gate-keeper particularly for incorrect strand migration and alignment in HR^29^. MMR-deficient DLD-1 cells were therefore expected to serve as a positive control for HR activation. Remarkably, the expression profiles of the HR/NHEJ genes in DLD-1 cells were extremely similar to those observed in TL^+^ CML cells or Ewing sarcoma cells, which may also suggest the possibility of the HR genes and, consequently, of the HR pathway being activated in these TL^+^ cells. In Fig. 1E, the expression levels of the *RAD51* gene are compared among several TL^+/−^ cell lines. The high levels of *RAD51* expression were most remarkable in TL^+^ hematogenic tumor cell lines, K562, Raji and Daudi, and also in MMR-defective colorectal cancer cell line, DLD-1. The two Ewing sarcoma cell lines, WE-68 and SK-N-MC, were next to them, although *RAD51* expression being relatively low in the latter. The expression levels in TL-negative cell lines were overall lower, which has however not been statistically confirmed. Thus, we have observed that expression of *RAD51* and the other HR genes was generally upregulated in TL^+^ cells, as in MMR-defective cells. RAD51 protein expression in these cell lines was confirmed, using a quantitative immunoblotting system (Fig. S1). Protein expression of the other HR/NHEJ pathway components was also confirmed (Fig. S2).

### RAD51 and 53BP1 foci are formed in Ewing sarcoma cells

**Figure 2:**
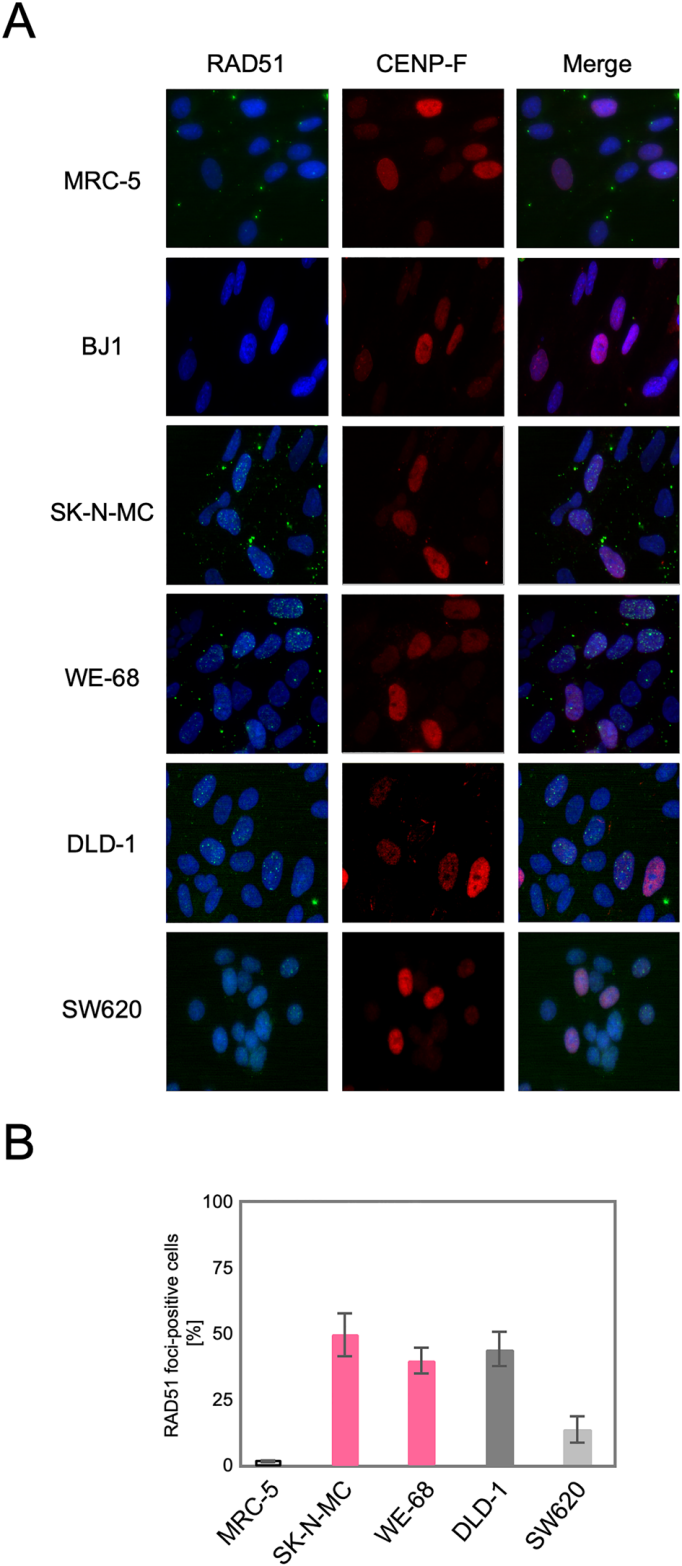
RAD51 foci formed in in SK-N-MC and WE-68 Ewing sarcoma cells. Exponentially growing cells were fixed with methanol and then reacted with the primary antibodies against RAD51 or CEMP-F, followed by the Alexa Fluor-labeled secondary antibodies. Nuclei were counterstained with DAPI. In each cell line, images were captured from at least ten visual fields using fluorescence microscope, and representative results are shown (**A**). RAD51 foci-positive cells were counted in ten visual fields (see also “Materials and methods” section). In three independent series of observations, the means and standard deviations were calculated. The results are compared in a bar chart (**B**).

**Figure 3:**
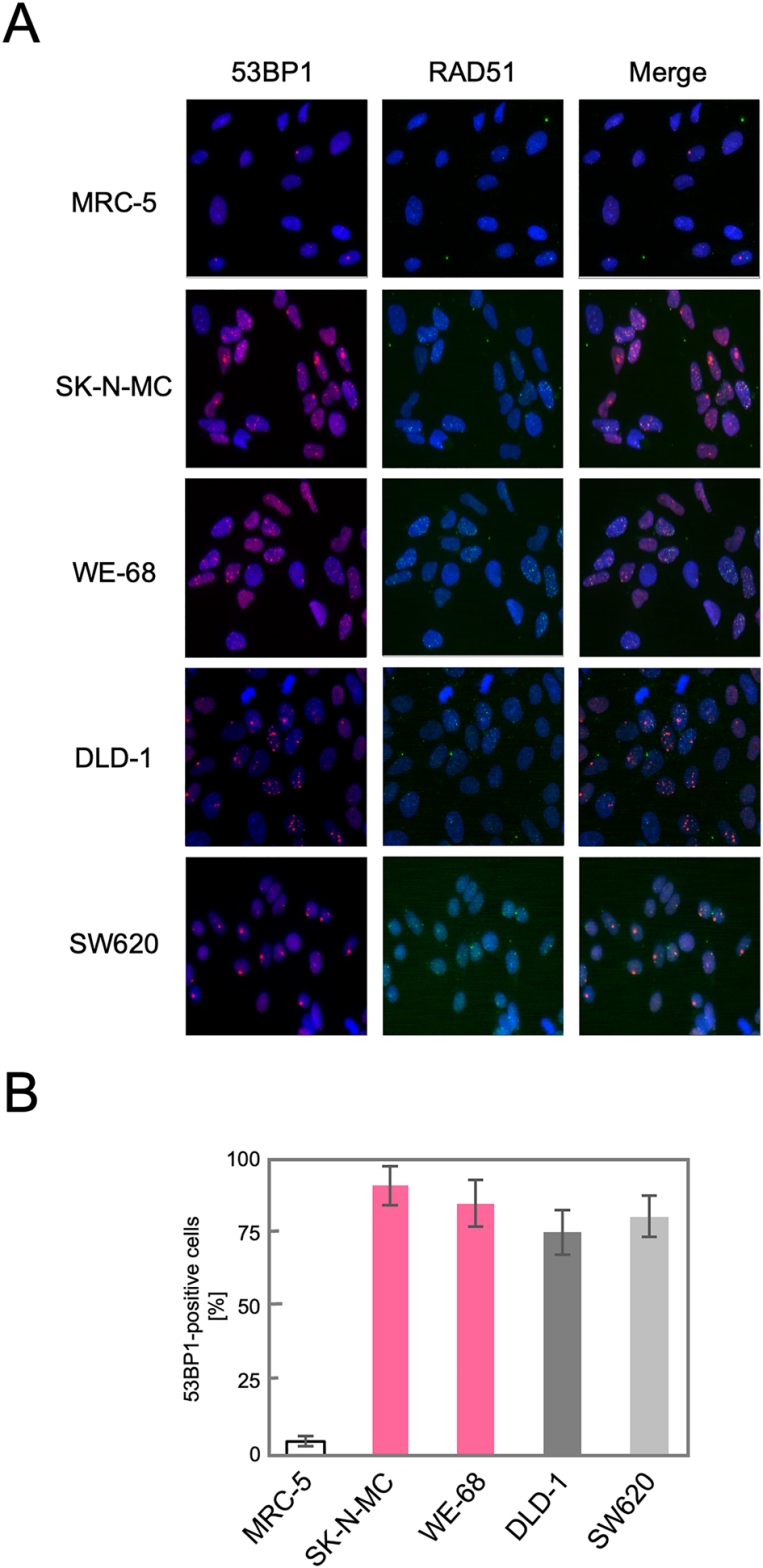
53BP1 co-localizes to RAD51 foci in Ewing sarcoma cells. 53BP1 foci were similarly assayed (see Fig. 2) (**A**). 53BP1 foci-positive cells were counted and compared (**B**).

**Figure 4:**
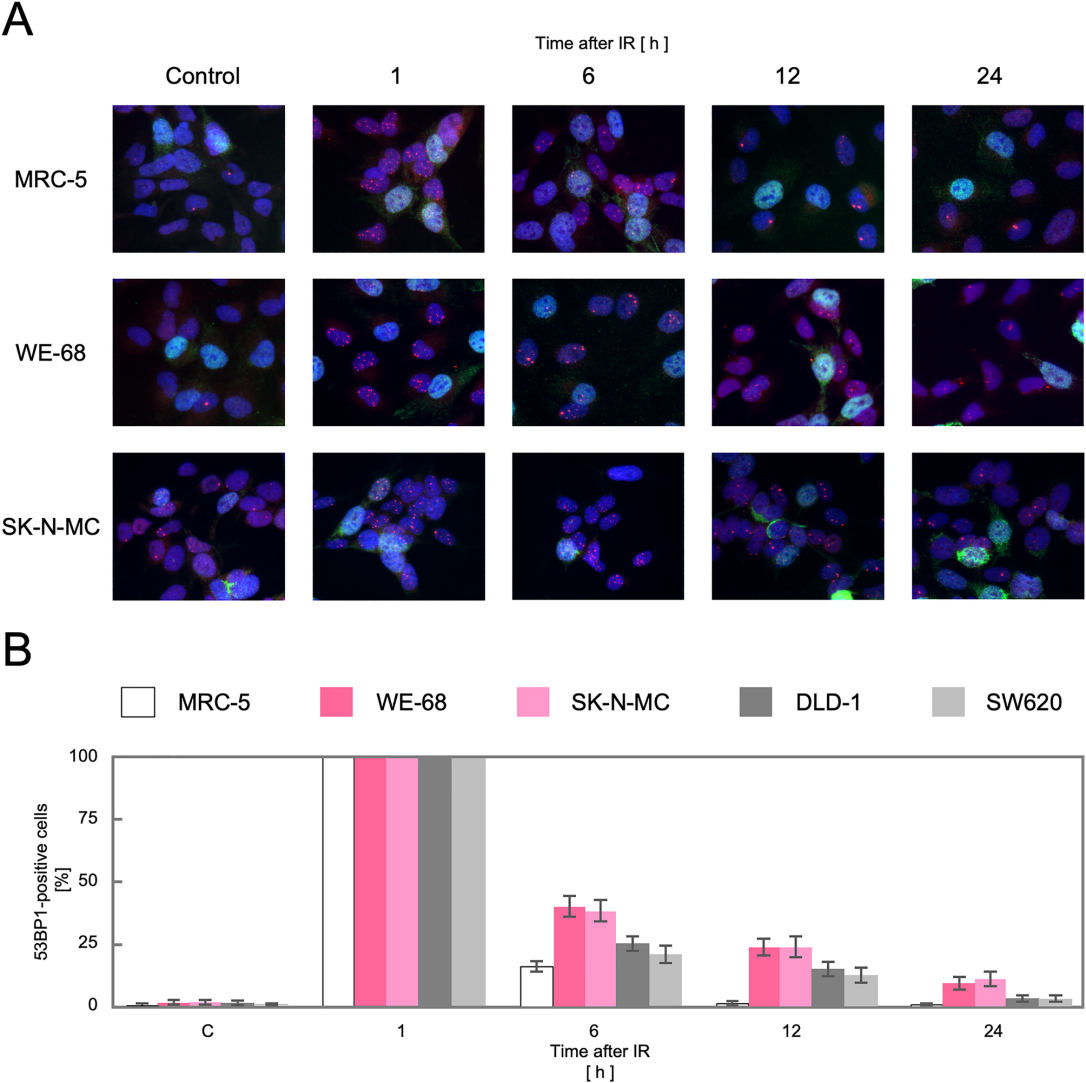
RAD51 foci observed after ionizing irradiation in Ewing sarcoma cells at G1 phase. Cultured cells were exposed to γ-rays, and 53BP1 focus formation in the time course has been observed. Representative images obtained in TL^+^ Ewing sarcoma cell lines, WE-68 and SK-N-MC, and the control, MRC-5, are shown (**A**). The relative levels of 53BP1-positive cells in each time point were plotted in a bar chart, with those at 1 h after irradiation being 100%. The means and standard deviations were calculated in three independent series of observations (**B**).

We next tested whether the upregulation of the HR genes indeed leads to an activation of the HR pathway in these TL^+^ cells. DSBs, caused either physiologically or artificially, induce the formation of nuclear foci comprised of multiple protein complexes functioning in the DNA repair pathways in cells. RAD51 forms a specific protein-DNA complex at the initial step of strand migration in the HR reactions^30^ and is one of the proteins that form nuclear foci in response to DSBs^31^. We therefore observed focus formation of RAD51 in various cell lines. As shown in Fig. 2A, RAD51 foci were extremely rare at the steady state in normal human fibroblast primary culture, MRC-5, and immortalized fibroblast cell line, BJ1, which implies that HR-processed and, presumably, overall DSBs induced intrinsically are at extremely low levels in these normal cells. The most remarkable finding is that numerous RAD51 foci were observed at the steady state in MMR-deficient DLD-1 cells whereas not in MMR-proficient SW620 cells. This is highly consistent with the above mentioned knowledge that the HR pathway is activated in MMR-defective cells. More remarkably, numerous RAD51 foci were similarly observed also in TL^+^ Ewing sarcoma cell lines, WE-68 and SK-N-MC (Fig. 2A). The number of RA51 foci-positive cells was compared among these cell lines (Fig. 2B), which clearly indicates that RAD51 focus formation in TL^+^ Ewing sarcoma cells is at the same level as in MMR-deficient DLD-1 cells. It may also be noteworthy that RAD51 foci-positive cells were significantly more frequent in the MMR-proficient SW620 cell line than in the control MRC-5, which may imply that HR-processed DSBs are more frequent on the cancer genome at the steady state. Their levels, however, appear much higher on the genomes of TL^+^ Ewing sarcoma cells. Predominance of HR-processed DSBs in Ewing sarcoma cells is also supported by our observations of 53BP1 foci in these cell lines. 53BP1 is a key DNA damage response factor that is recruited to the DSB sites at an early phase of DSB repair and plays a critical role in pathway switching between NHEJ and HR^32^. Similar focus assays revealed that 53BP1 foci were abundant in all the cell lines except the normal control, MRC-5, and that they were co-localized to RAD51 foci (Fig. 3. See also Fig. S3). One remarkable difference between 53BP1 and RAD51 foci is that the level of 53BP1 foci was high in MMR-proficient SW620 cells in which RAD51 foci were less frequent. This observation may suggest the possibility that DSB repair pathway be switched to the HR pathway in TL^+^ or MMR-deficient cells whereas in MMR-proficient cells to NHEJ. In order to pursue the difference in DSB repair activities between MMR-deficient and TL^+^ cells, we next observed 53BP1 foci formed after ionizing irradiation. As shown in Fig. 4, 53BP1 foci remarkably increased after irradiation and thereafter decreased time-dependently. In particular, the foci disappeared most rapidly in MRC-5 cells, reflecting their normal and active DSB repair. An intriguing difference between TL^+^ and MMR-deficient cells is the rate in decline of 53BP1 foci after irradiation. It is clearly shown in Fig. 4B that the decrease of 53BP1 foci was significantly retarded in TL^+^ WE-68 and SK-N-MC cell lines, which may suggest an imbalance between the two pathways in DSB repair in these TL^+^ cells. In WE-68 or SK-N-MC cells, the NHEJ pathway may be less active for unknown reasons and the HR pathway may, consequently, be activated.

### Chromosome aberrations induced by fork stress in Ewing sarcoma cells

**Figure 5:**
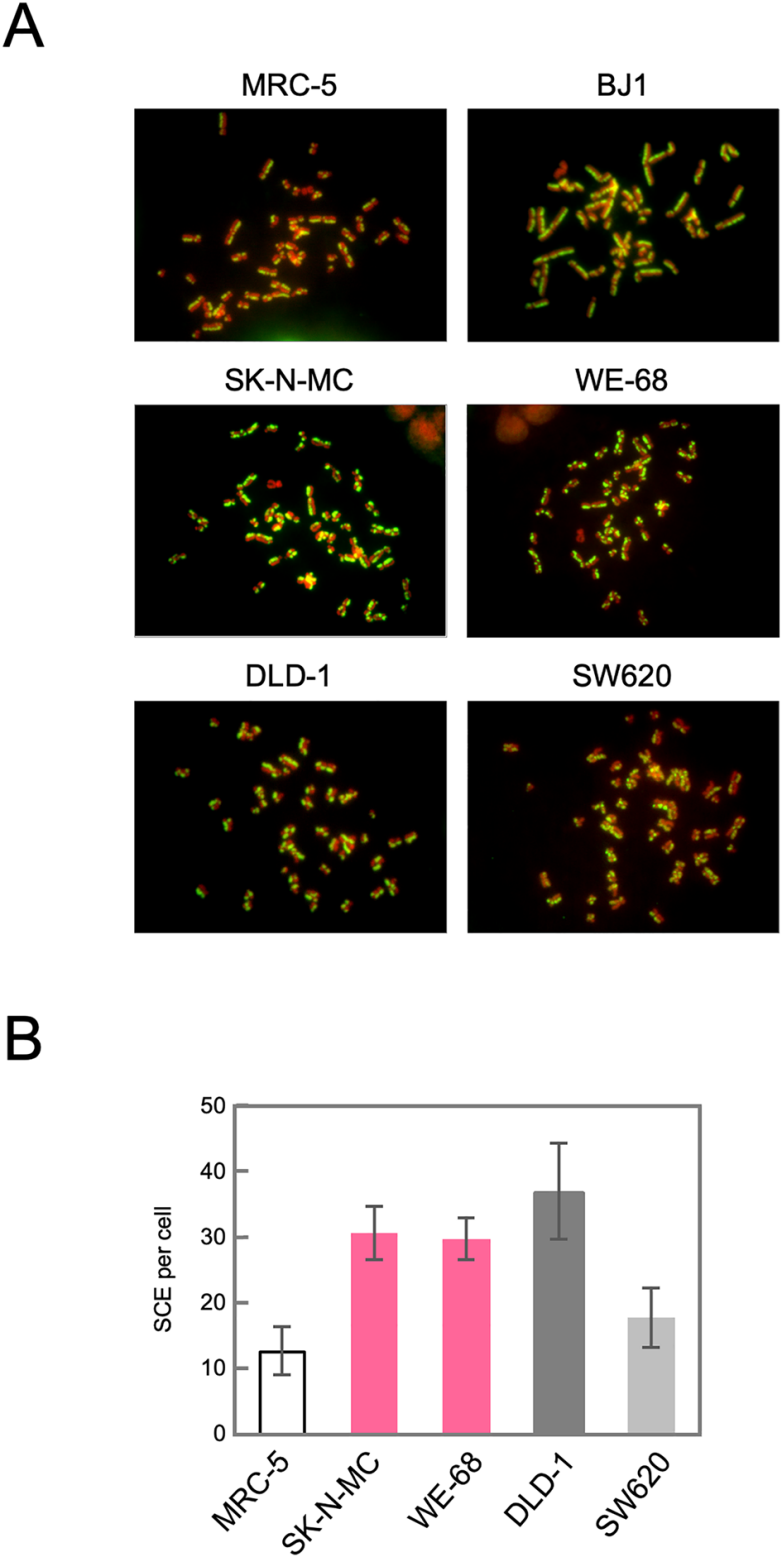
SCE induced after EdU treatment in Ewing sarcoma cells. Exponentially growing cells were treated with 1 µM EdU for 24 h. Then, metaphase cells were collected, and chromosomes were spread onto a slide glass, which was then subjected to the EdU detection according to the manufacturer’s protocol and stained with DAPI. Images were captured using fluorescence microscope, and representative results are shown (**A**). SCE was counted in 20 independent metaphase cells. The means and standard deviations were calculated and shown in a bar chart (**B**).

The activation of the HR pathway in TL^+^ Ewing sarcoma cell lines has thus been suggested, in terms of gene expression and subcellular protein localization. To test whether these observed phenomena are relevant and indeed lead to structural outcomes in the genomic DNA, we next focused on chromosomal aberration dependent on the HR pathway. Sister chromatid exchange (SCE) is a phenomenon known as reflecting an incorrect resolution of Holliday structures formed in the intermediate process of HR in S-phase cells. Since it is possible to introduce strand breaks into S-phase cells by drug induced fork stress, we treated cells with EdU and observed the SCE phenomenon induced in each cell line (Fig. 5). SCE was most frequently observed in MMR-deficient DLD-1 cells, which is highly compatible with their potent HR activities and forms a clear contrast to MMR-proficient SW620 cells. SCE in TL^+^ WE-68 and SK-N-MC cells was at a similar level to DLD-1, which indicates that SCE frequently occurs in response to DSBs caused by fork stress in the S-phase reflecting the active HR pathway in these cells. Thus, we have also shown the functional aspects of HR activation in TL^+^ Ewing sarcoma cells.

## Discussion

The problems in approaching TL mechanisms may be classified into those related to *cis* elements and those to (a) *trans*-acting factors, the former being further divided into problems of (b) chromosomes and of (c) DNA sequences. TL mechanisms have thus far been approached chiefly from the viewpoints of *cis* elements. The *trans*-acting factors, i.e. the proteins functioning in the DSB repair pathways, however, have not been intensively approached and have first been systematically addressed in our present study. The pathway switching in DSB repair in mammalian cells is now widely discussed, and the balance between the two major repair modes, i.e. HR and NHEJ, is currently regarded as depending primarily on cell cycle phase, chromatin topology and subnuclear localization, which implies that the activities of the two pathways are physiologically variable and variously regulated depending on the biological states of cells. Although it is widely known that NHEJ predominates in mammalian DSB repair whereas HR does in yeasts^33^, the counterbalance between HR and NHEJ is obviously not unchangeable. In our present study, the predominance of the HR pathway activities has been shown in TL^+^ Ewing sarcoma cells, in terms of gene expression, subcellular protein localization and chromosome alterations. Although our observations were limited and NHEJ proteins were not directly addressed, these findings may suggest the involvement of HR in TL mechanisms. One important observation in this study is the discrepancy in focus formation between 53BP1 and RAD51 in SW620 cells (Figs. 2, 3). In this MMR-proficient cancer cell line, the level of 53BP1 foci was high, similarly to MMR-deficient or TL^+^ Ewing sarcoma cell lines, whereas RAD51 foci were obviously less frequent, which implies that DSBs caused in SW620 cells are at a level similar to those in the other cell lines, and that the HR-processed fraction is however relatively limited. In other words, DSB repair pathway switching may be biased to the NHEJ pathway in SW620 cells, whereas in TL^+^ or MMR-deficient cells to HR. Furthermore, the balance between HR and NHEJ is also unphysiologically, i.e. pathologically, variable. The HR and NHEJ pathways include many subcomponents that are compromised in human neoplasms or in inherited predispositions to cancer. Typical examples are HBOC (*BRCA1*^21^/*2*^22^) and its related cancer susceptibilities designated as ‘HR-deficiency (HRD)’(*RAD50*^34^, *RAD51*^35^, *RAD51C*^36,37^/*D*^38^*, BARD1*^39^*, BRIP1*^40^*, PALB2*^41^), Nijmegen breakage syndrome (*NBS1*^42^) and LIG4 syndrome (*LIG4*^43^). The existence of these pathological states clearly implies that the activities of the HR and NHEJ pathways are sometimes compromised in human cells. Indeed, as shown in Fig. 4B, the decrease of 53BP1 foci after ionizing irradiation was significantly retarded in TL^+^ cell lines compared with the normal control, and the rate in decline of 53BP1 foci was lower than in MMR-deficient lines, which may suggest that the NHEJ pathway is less active in TL^+^ Ewing sarcoma cells, although the abnormalities in the NHEJ genes are currently not known in these TL^+^ cell lines and, on the contrary, compromised HR has also been reported in Ewing sarcoma cells^44^. We thus point out an imbalance between the HR and NHEJ pathways in TL^+^ Ewing sarcoma cells. In these cells, the HR pathway is highly activated whereas the NHEJ pathway may be significantly compromised, although due to technical limitations we could not show the latter directly. On the other hand, in MMR-deficient cells, the former is similarly activated, whereas the latter may be intact.

**Figure 6:**
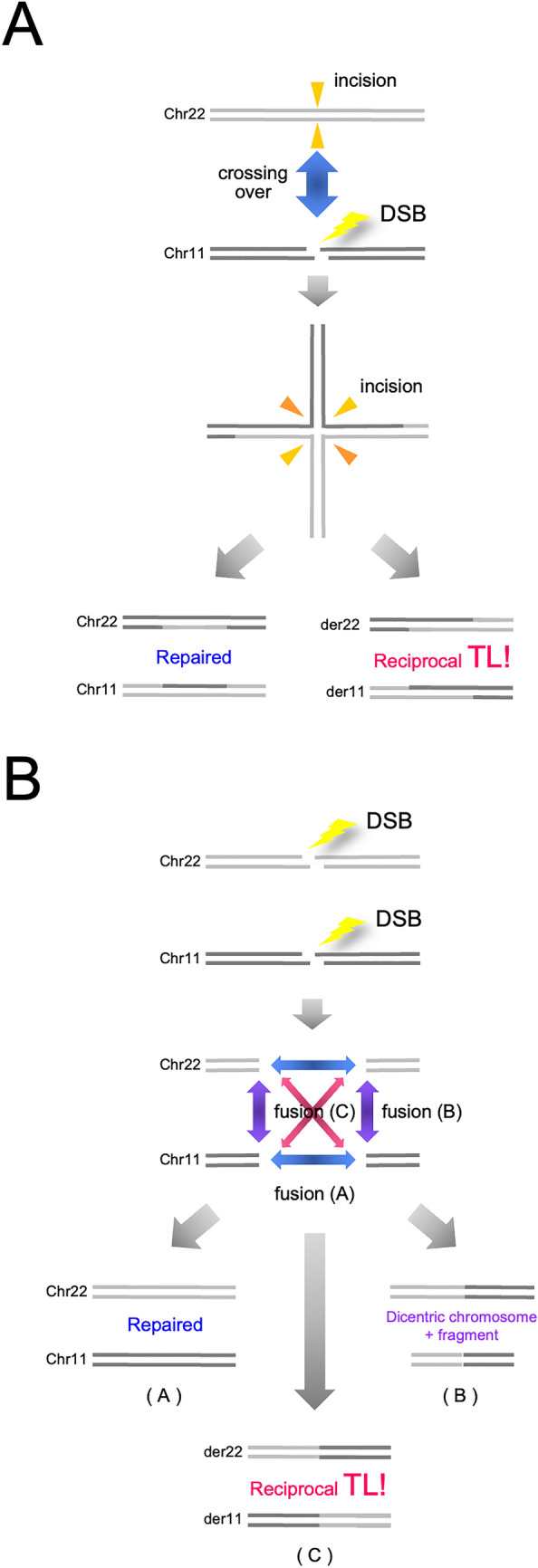
HR and NHEJ models for chromosome translocation. The HR (**A**) and NHEJ (**B**) models to explain t(11;22) chromosome translocation are schematized. In the HR model, one DSB is required in either chromosome, and TL is obtained with a 1/2 chance. On the other hand, in the NHEJ model, two DSBs are necessary in both chromosomes, and the probability of reciprocal TL is 1/3. The results also include dicentric chromosomes and fragments.

The *cis* elements in TL mechanisms have thus far been actively approached and discussed. Since recombinational models were initially proposed, recombinogenic sequences have been intensively explored in the genomic regions encompassing the breakpoints. One important trait in the distribution of these sequences is that the sequence categories differ depending on the disease. In the CML case, Alu-homology and chi-consensus sequences were found in the vicinity of the breakpoints^18^. On the other hand, chi-like sequences distribute in the breakpoint regions in case of malignant lymphomas carrying t(14;18) or t(8;14) TL^11^, which leads to a transcriptional activation of *BCL2* or *MYC* by the intronic enhancer in the *IGH* locus^45^. In some subtypes of leukemia, the breakpoint cluster regions contain topoisomerase II consensus sequences^10^, and purine-pyrimidine tracts forming Z-DNA were found in t(11;14) TL^+^ lymphoid tumors^16^. However, the most recurrent recombinogenic sequences associated with the TL breakpoints are a SINE type repeat, Alu. Indeed, Alu sequence subtypes were abundantly identified in the genomic regions that encompass TL junctions in Ewing sarcomas^23^. Thus, recombinogenic sequence homology has been confirmed in this TL^+^ neoplasm. Indeed, HR, rather than NHEJ mechanisms, may well explain reciprocal and balanced TL, because the end joining of the two distant chromosome contexts is simply explicable by pivotal bidirectional resolution of Holiday structures whereas NHEJ requires a model to explain multicentric DSBs and selective end joining of them (Fig. 6). Since Brunet E and colleagues have artificially reconstructed *EWS*-*FLI1* fusion genes in vitro by concurrently introducing two DSBs into cells using the sequence-specific genome editing techniques^24^, the c-NHEJ pathway has been regarded as the mechanism explaining t(11; 22) TL and the *EWS*-*FLI1* fusion gene. However, four persistently co-existing DNA ends in cells may be fused to form t(11;22) TL with a 1/3 chance (Fig. 6), and two DSBs were selectively introduced in the two genetic loci in this system. This selectiveness is an essential problem and also needs to be addressed in HR models. One possible explanation is the proximity between two genetic loci or chromosome territories. The intranuclear spaces allocated to each chromosome in the interphase are designated as ‘chromosome territory’^19,46^. Two genomic regions proximately arranged in nuclei may increase the chance to cross over. This problem has been cytogenetically approached using interphase FISH. Although the territories of chromosome 9 and 22 were not shown to be similar^19^, Kozubek and colleagues have demonstrated that the *ABL* and *BCR* loci are proximately distributed in the interphase nuclei in some subpopulations of lymphocytes or bone marrow cells^20,47^. However, they also found that the distance between the two loci was different depending the biological conditions^20,47^. On the other hand, they similarly analyzed the distance between the *EWS* and *FLI1* loci in lymphocytes, which, they concluded, was larger than that between *BCR* and *ABL*^48^, although the precursor of Ewing sarcoma cells is not of lymphoid lineage. The proximity in chromosome positioning may be a promoting, but not necessarily prerequisite condition for TL. Another problem in the HR model is the Alu homology. Alu sequences are highly polymorphic and, consequently, not mutually identical. In other words, they are not completely homologous. When crossing over occurs between two independent Alu repeats, sequence mismatches may activate MMR and, consequently, abrogate the HR reactions. In this point of view, HR activation in MMR-defective cells is noteworthy. In order to test the hypothesis of compromised MMR in Ewing sarcoma cells, we therefore analyzed microsatellite stability in the two cell lines, WE-68 and SK-N-MC. However, destabilization of microsatellites was not observed (Fig. S4 and S5), which implies that MMR is proficient at least in these two cell lines. The crossing over between incompletely homologous sequences in MMR-proficient cells remains as an enigmatic problem.

## Materials and methods

### Chemicals

All chemicals were purchased from Sigma-Aldrich (St. Louis, MO, USA) unless otherwise indicated.

### Cell culture

Normal human diploid fibroblast primary culture, MRC-5 and cell line, BJ1-hTERT, were obtained from CLONTECH Lab Inc. (Palo Alto, CA, USA), and Japanese Collection of Research Bioresources (JCRB) Cell Bank (Tokyo, Japan), respectively. They were cultured in minimum essential media (MEM) supplemented with 10% fetal bovine serum (FBS) (Gibco, Thermo Fisher Scientific, Waltham, MA, USA).

Human Ewing sarcoma cell lines, RD-ES, SK-ES-1, and SK-N-MC were obtained from the American Type Culture Collection (Manassas, VA, USA). These cells were maintained in Dulbecco's modified Eagle's medium (DMEM) supplemented with 10% FBS and 1% penicillin and streptomycin. Human Ewing sarcoma cell line, SCCH-196, was obtained from JCRB Cell Bank and maintained in MEM (Gibco, Thermo Fisher Scientific) supplemented with 10% FBS and 0.1 mmol/l nonessential amino acids (NEAA) (Gibco, Thermo Fisher Scientific). Human Ewing sarcoma cell line, WE-68, was kindly provided by Dr Frans van Valen (Westfalische-Wilhelms University, Münster, Germany) and was maintained in RPMI1640 (Gibco, Thermo Fisher Scientific) supplemented with 10% FBS. Human osteosarcoma cell lines, MG63, HOS and Saos2, were obtained from RIKEN cell bank (Tsukuba, Japan), and NY and Hu09 from JCRB Cell Bank. MG-63, Saos2 and NY being maintained in DMEM supplemented with 10% FBS, 100 µg/mL penicillin and 100 µg/mL streptomycin, whereas HOS being maintained in MEM supplemented with 10% FBS and 0.1 mmol/L NEAA and Hu09 in RPMI1640 supplemented with 10% FBS. The above cells were cultured at 37 °C and 5% CO_2_.

DLD-1 and SW620 were obtained from American Type Cell Culture. DLD-1 and SW620 cells were maintained in RPMI1640 and Leibovitz’s L-15 media (Gibco, Thermo Fisher Scientific), respectively. The media were supplemented with 10% fetal bovine serum (Sigma-Aldrich), 100 units/mL penicillin and 100 μg/mL streptomycin (Gibco, Thermo Fisher Scientific). DLD-1 cells were cultured at 37 °C and 5% CO_2_, whereas SW620 cells were kept under room air.

### γ-irradiation of cells

Cells cultured in a culture flask were exposed to γ-rays from a γ-ray irradiator equipped with a ^137^Cs source (Pony Industry Co., Ltd, Osaka, Japan) at a dose rate of 1 Gy/min.

### Expression microarray analyses

mRNA expression in Ewing sarcoma, osteosarcoma, and colon cancer cell lines was profiled using GeneChip Genome HG U133 Plus 2.0 Array (Affymetrix, Thermo Fisher Scientific, Santa Clara, CA, USA), according to the manufacture’s protocols. Briefly, total RNA was extracted from cells and its quality was confirmed using Bioanalyzer 2100 (Agilent, Santa Clara, CA). Reference RNA were also obtained from Takara Bio Inc. (Kusatsu, Japan). Double stranded cDNA was synthesized using 1 ng of total RNA by reverse transcription with a T7 promoter-tailed oligo dT primer. Biotinylated-cRNA was then synthesized using the 3’ IVT Express Kit (Affymetrix, Thermo Fisher Scientific), according to the manufacturer’s protocols. After fragmentation, 12.5 μg of the biotinylated-cRNA were hybridized to the expression array for 16 h. The GeneChip arrays were stained using GeneChip Fluidics Station 450 (Affymetrix, Thermo Fisher Scientific) and scanned using GeneChip Scanner 3000 (Affymetrix, Thermo Fisher Scientific), according to the manufacture’s protocols. The numerical values of GeneChip arrays were analyzed using GeneSpring GX 11.0 software for normalization and filtering by the normalized data (20.0–100.0th‰). Analysis of variance was carried out to determine significant difference between two groups.

### Immunofluorescence staining

Exponentially growing cells were collected by trypsinization, and 5 × 10^4^ cells were replated onto coverslips. The cells were fixed with cold methanol for 10 min on ice, followed by washing with 1 × PBS. Then, the primary antibodies diluted in TBS-DT (20 mM Tris–HCl, pH7.6, 137 mM NaCl, 0.1% Tween 20, 125 µg/mL ampicillin, 5% skim milk) were treated for 2 h at 37 °C, followed by the Alexa Fluor-labeled secondary antibodies for 1 h at 37 °C. Nuclei were counterstained with 1 µg/mL DAPI. The antibodies used were anti-RAD51 (14B4, GTX70230, GeneTex, Irvine, CA, USA), anti-CENP-F (NB500-101, Novus Biologicals, Centennial, CO, USA), anti-53BP1 (A300-272A, BETHYL Laboratories, Inc., Montgomery, TX, USA), Alexa Fluor 488-labeled anti-mouse IgG (A11001, Thermo Fisher Scientific), and Alexa Fluor 555-labed anti-rabbit IgG (A21428, Thermo Fisher Scientific). Images were captured by fluorescence microscope (DM6000B, Leica Japan, Tokyo, Japan) and analyzed by FW4000 (Leica Japan). Fluorescence-positive foci were defined as those with signals two times higher than the background levels, and foci-positive cells as those with at least one fluorescence-positive focus. The observations were done in at least three independent series.

### Immunoblotting

Exponentiall growing cells were collected by trypsinization, washed with PBS, and lysed in RIPA buffer (50 mM Tris–HCl, pH 7.2, 150 mM NaCl, 1% Nonidet P-40, 1% Sodium deoxycholate and 0.1% Sodium dodecylsulfate) containing protease inhibitor cocktail (Roche Japan, Tokyo, Japan). Protein concentrations were determined by BCA protein assay (Thermo Fisher Scientific), and 8 µg of proteins were electrophoresed on 5–10% SDS–polyacrylamide gels, and then electrophoretically transferred to a polyvinyl difluoride membrane. Primary antibodies used in this study are as follows: anti-RAD51 (GTX70230, GeneTex), anti-MRE11 (clone 12D7, GeneTex), anti-ATM (clone 1A1, GeneTex), anti-Rad50 (clone 13B3, GeneTex), anti-NBS1 (clone 1D7, GeneTex), anti-BRCA1 (clone 6B4, GeneTex), anti-BRCA2 (clone 1B8, GeneTex), anti-Ku86 (clone 111, Kamiya Biochemical co., Seattle, WA, USA), anti-Ku70 (clone N3H10, Kamiya Biochemical co.), anti-DNA-PKcs (MC-365, Kamiya Biochemical co.), anti-XRCC4 (GTX70293, GeneTex), anti-Lig IV (GTX74360, GeneTex), anti-XLF (A300-730, BETHYL Laboratories, Inc.), and anti-γ-tubulin (clone DTU-88, Sigma-Aldrich).

### Microsatellite instability

High-resolution multi-fluorescence microsatellite analysis has been described elsewhere in detail^49–51^. Briefly, microsatellite sequences were amplified by PCR using *TaKaRa Taq* (Takara Bio Inc.) and primers labelled with a fluorescent dye, 6-FAM™ (6-carboxyfluorescein) or HEX™ (6-carboxy-2′,4′,7′,4,7,hexachloro-fluorescein) (Applied Biosystems, Thermo Fisher Scientific, Foster city, CA, USA). PCR products were electrophoresed in the ABI PRISM™ 310 Genetic Analyzer (Applied Biosystems, Thermo Fisher Scientific), and the data were processed using GeneScan™ Software v3.1.2 or GeneMapper™ Software v4.1 (Applied Biosystems, Thermo Fisher Scientific). In each assay, the electrophoretic profiles of two (and sometimes more) samples, which include controls, were compared. For the comparison, two PCR products independently labelled with different fluorescent dyes were mixed and run in the sequencer, or two profiles of independent runs were merged using the softwares.

### Sister chromatid exchange

Exponentially growing cells were incubated with a growth medium containing 1 µM EdU for 24 h. Then, metaphase cells were collected by trypsinization. After centrifugation at 1200 rpm for 5 min, cell pellets were reconstituted and treated with hypotonic buffer (0.075 M KCl) for 20 min at room temperature, followed by the fixation with Carnoy’s fixative (methanol:acetic acid = 3:1) on ice for 30 min. After fixation, cells were resuspended with appropriate volume of new Carnoy’s fixative, and the cell suspension was dropped onto a 70%-ethanol-immersed slide glass. The slide glass was dried for at least 1 day, and was subjected to the EdU detection according to the protocol provided by the manufacturer (C10340, Thermo Fisher Scientific). Chromosome samples were then stained with 1 µg/mL DAPI, and images were captured by fluorescence microscope (DM6000B, Leica Japan).

## Supplementary Information

Supplementary Information 1.

Supplementary Information 2.

## Data Availability

The datasets generated and/or analyzed in the current study are available from the corresponding author on reasonable request. Gene expression data were deposited as the accession number GSE70827 in http://www.ncbi.nlm.nih.gov/geo/query/acc.cgi?acc=GSE70826.
