# Supplementary material for: Activation of recombinational repair in Ewing sarcoma cells carrying EWS-FLI1 fusion gene by chromosome translocation
Source: Sci Rep. 2022 Aug 30;12:14764. doi: 10.1038/s41598-022-19164-x (PMC9427769; doi:10.1038/s41598-022-19164-x)
Supplement: Supplementary file 1 — Supplementary Information 1. [file 41598_2022_19164_MOESM1_ESM.pdf]

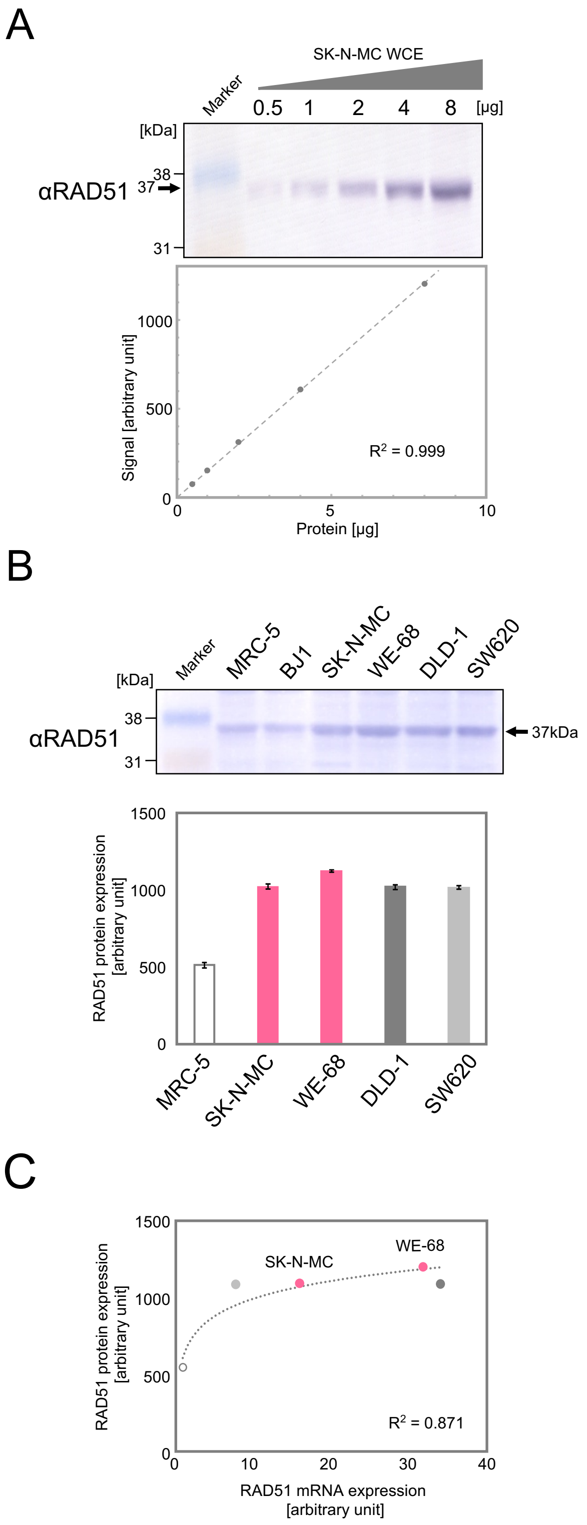

Fig. S1. RAD51 expression in TL$^+$ SK-N-MC and WE-68 Ewing sarcoma cells.
Expression of the RAD51 protein in cells was quantitatively assessed by immunoblotting. Detection of the RAD51 antigen was linear (R^2 = 0.99) in this system (A). RAD51 protein expression was compared among the six representative cell lines (for details, see Materials and Methods) (B). An approximately linear (R^2 = 0.87) relationship was observed between RAD51 protein and mRNA expression (C).

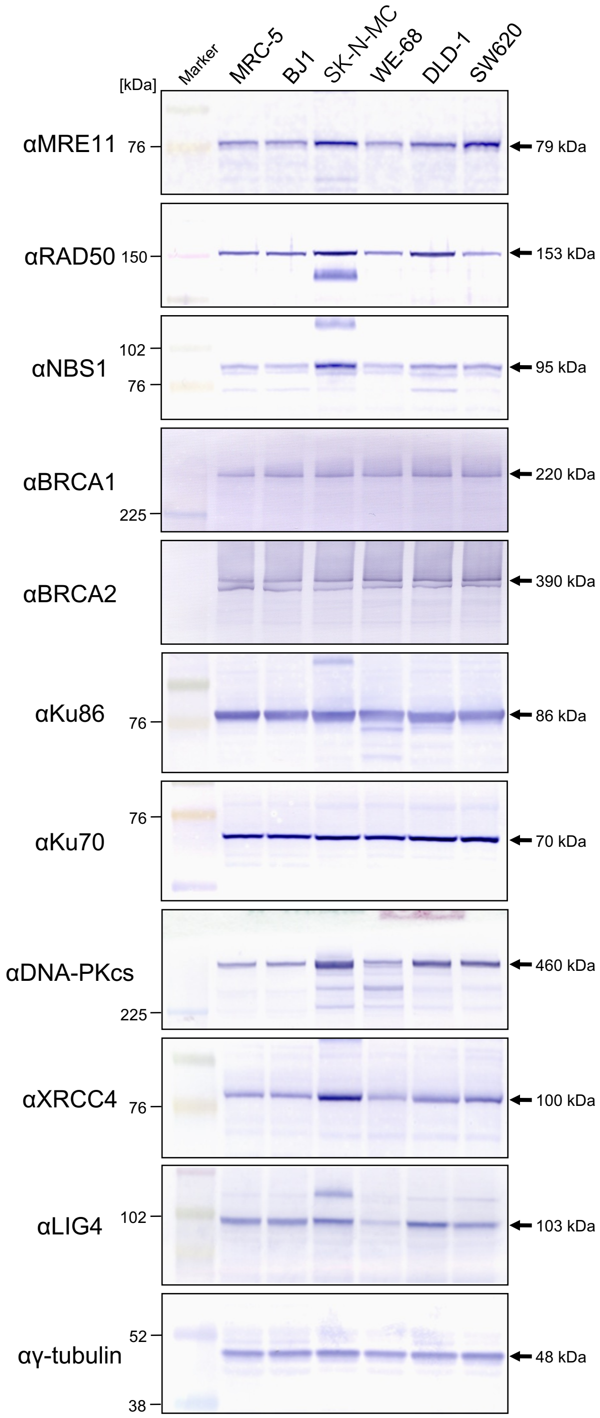

Fig. S2. Expression of the DSBR pathway components in SK-N-MC and WE-68 cells.

Expression of the proteins functioning in the DSBR pathways was observed using immunoblotting (for details, see Materials and Methods).

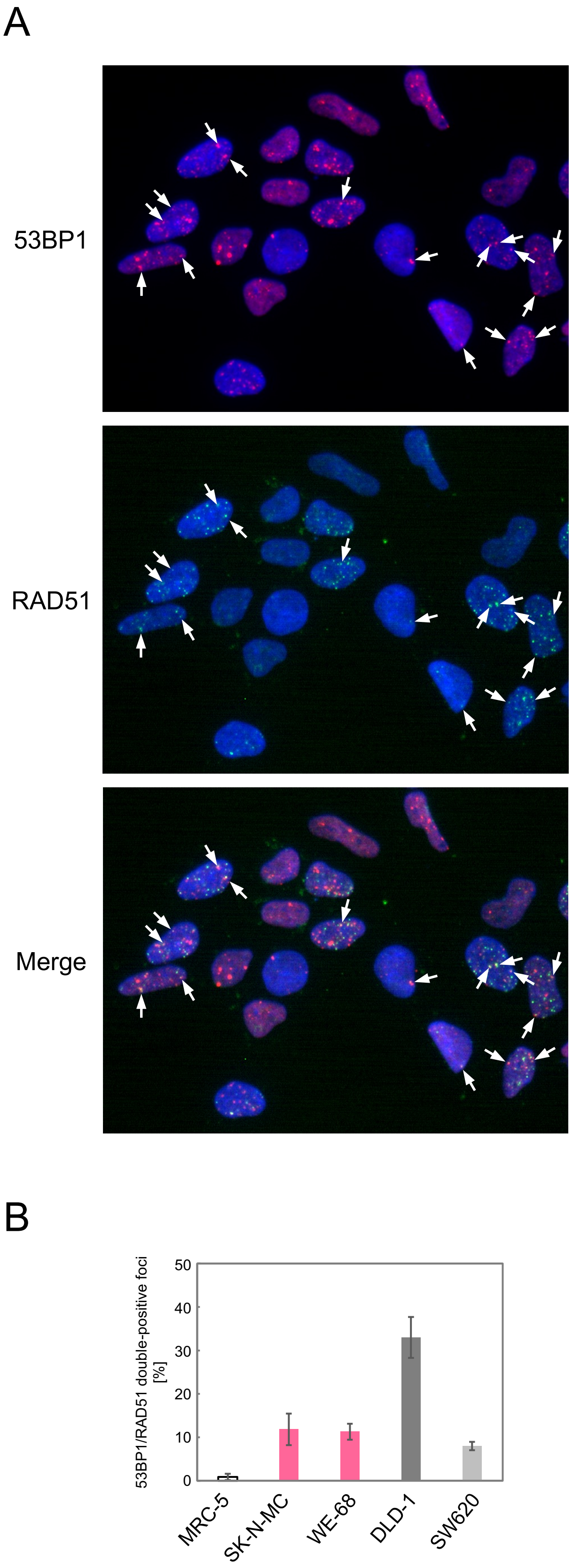

Fig. S3. Co-localization of 53BP1 and RAD51 in Ewing sarcoma cells.
In the magnified images of those shown in Figure 3A, 53BP1, RAD51 and 53BP1/RAD51 double positive foci in WE-68 cells, as an example, are indicated with white arrows (A). In each cell line, the ratio of 53BP1/RAD51 double -positive foci to all observed 53BP1 foci was calculated, and the results are shown in a bar chart.

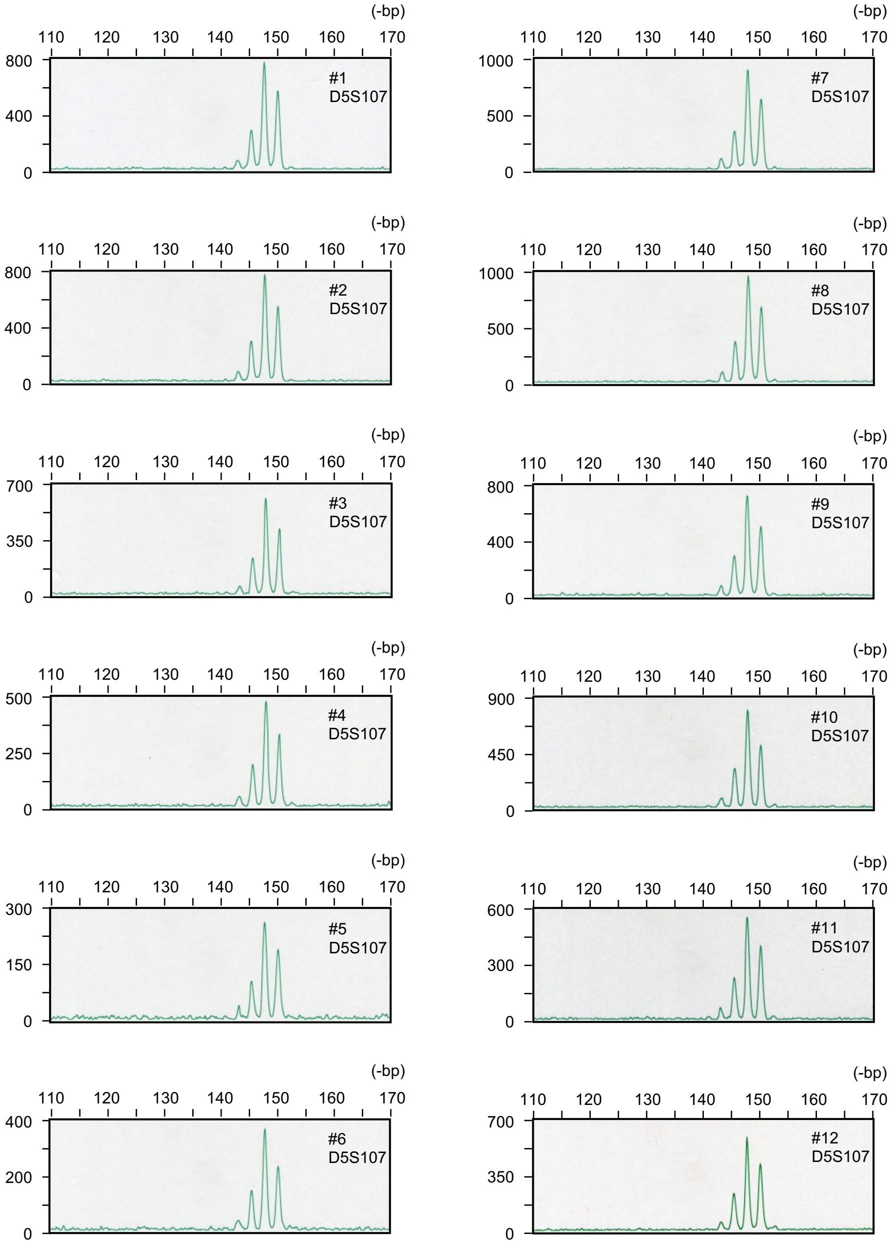

Fig. S4. Stable dinucleotide microsatellites in WE-68 cells.
High-resolution microsatellite analysis (see Materials and Methods) was done using subclones isolated from the WE-68 cell lines. In each subclone, several dinucleotide microsatellite sequence was analyzed. Results are compared among twelve subclones, and the representative results obtained in the D5S107 locus are shown. The x-axis corresponds to the fragment length, and the y axis to the amount of DNA fragments. Subclone names are indicated in each panel.

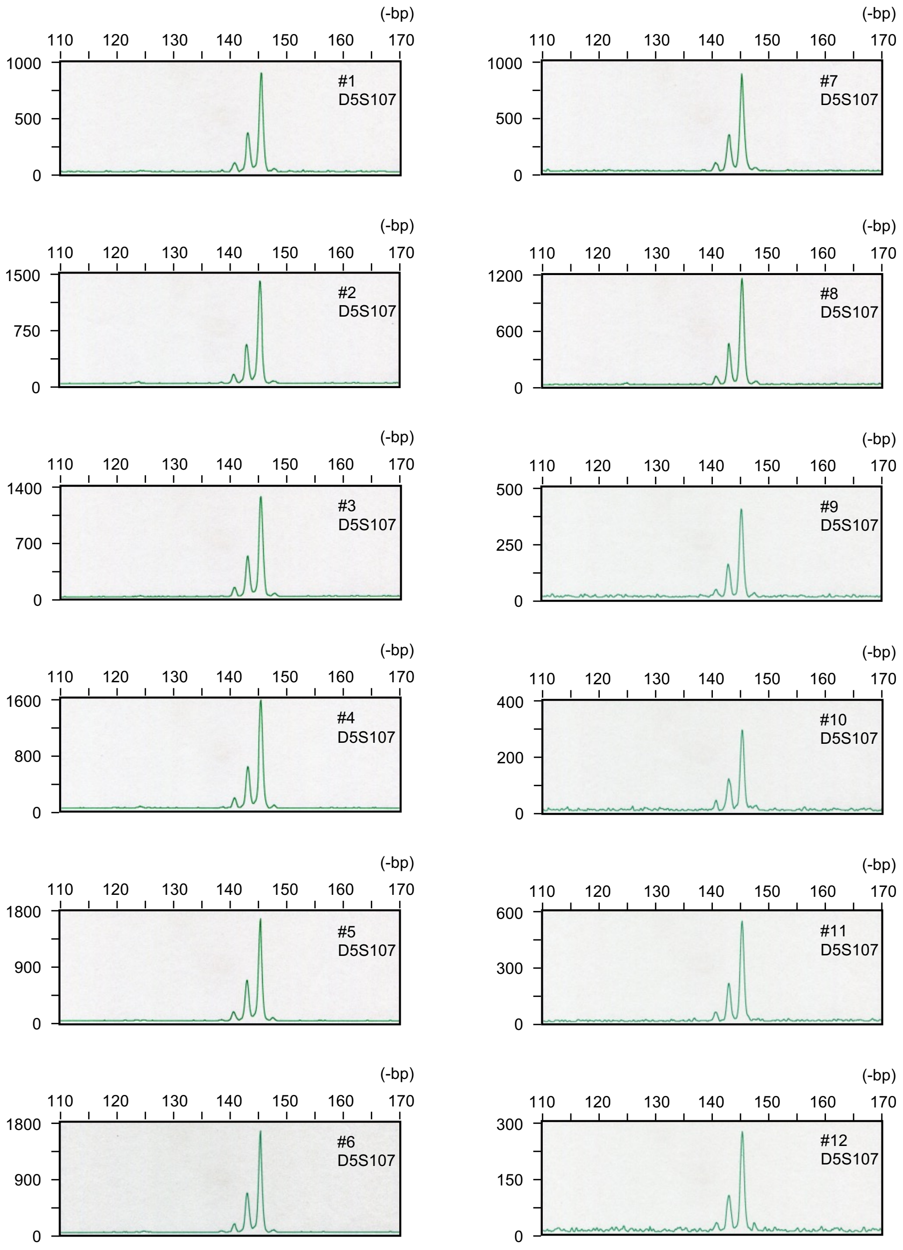

Fig. S5. Stable dinucleotide microsatellites in SK-N-MC cells.
High-resolution microsatellite analysis (see Materials and Methods) was similarly done using subclones isolated from the SK-N-MC cell lines. The representative results obtained in the D5S107 locus are shown. The x- and y-axes correspond to the length and the amount of DNA fragments, respectively. Subclone names are indicated in each panel.
