# Supplementary material for: Activation of recombinational repair in Ewing sarcoma cells carrying EWS-FLI1 fusion gene by chromosome translocation
Source: Sci Rep. 2022 Aug 30;12:14764. doi: 10.1038/s41598-022-19164-x (PMC9427769; doi:10.1038/s41598-022-19164-x)
Supplement: Supplementary file 2 — Supplementary Information 2. [file 41598_2022_19164_MOESM2_ESM.pdf]

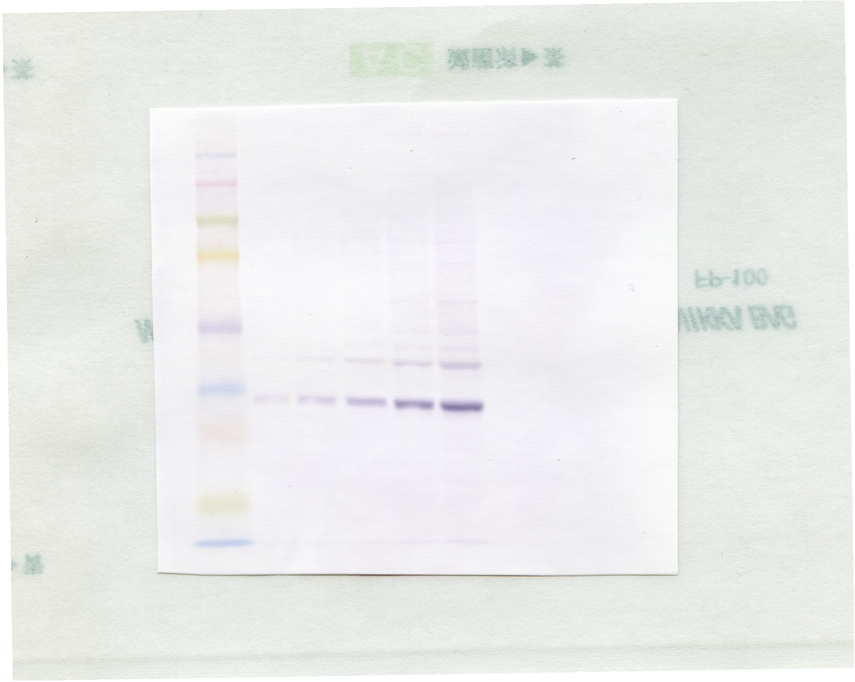

Fig. S1A. uncropped

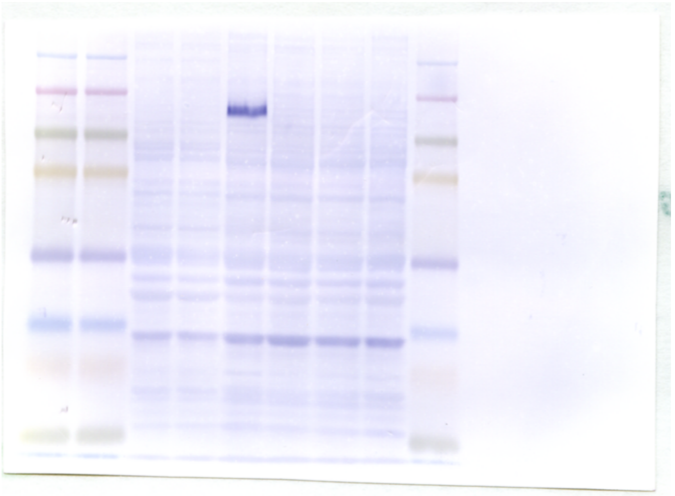

Fig. S1B. uncropped

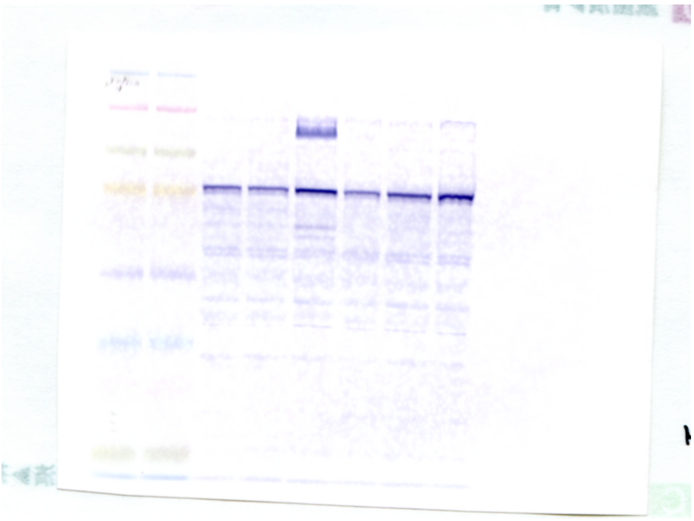

Fig. S2. MRE11. uncropped

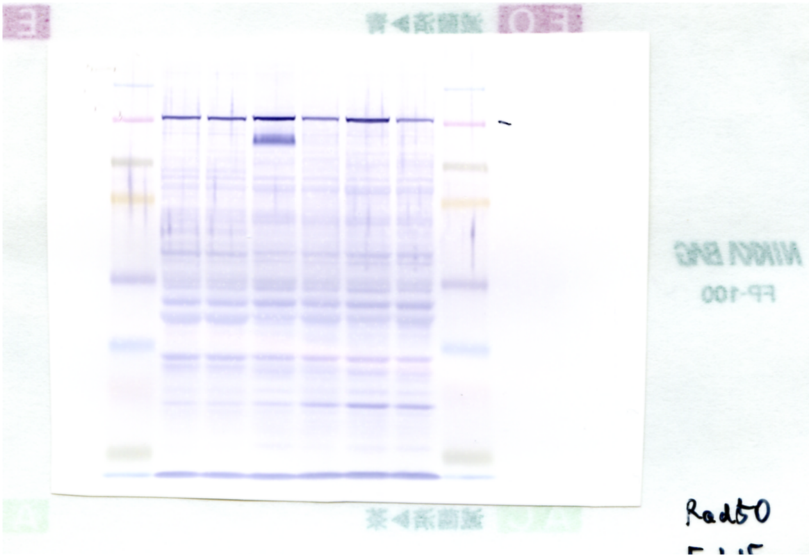

Fig. S2. RAD50. uncropped

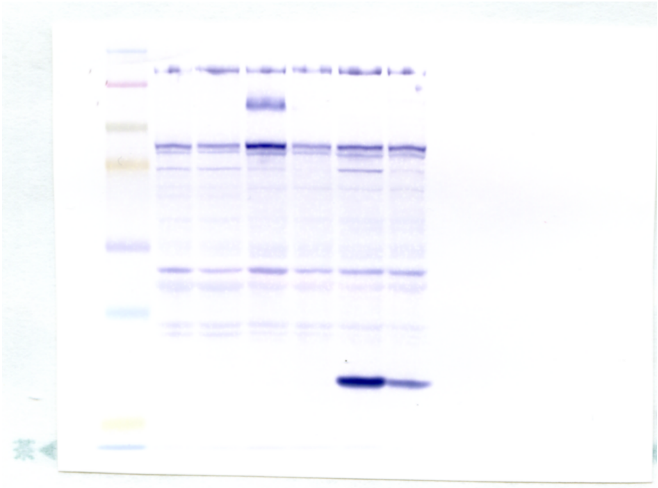

Fig. S2. NBS1. uncropped

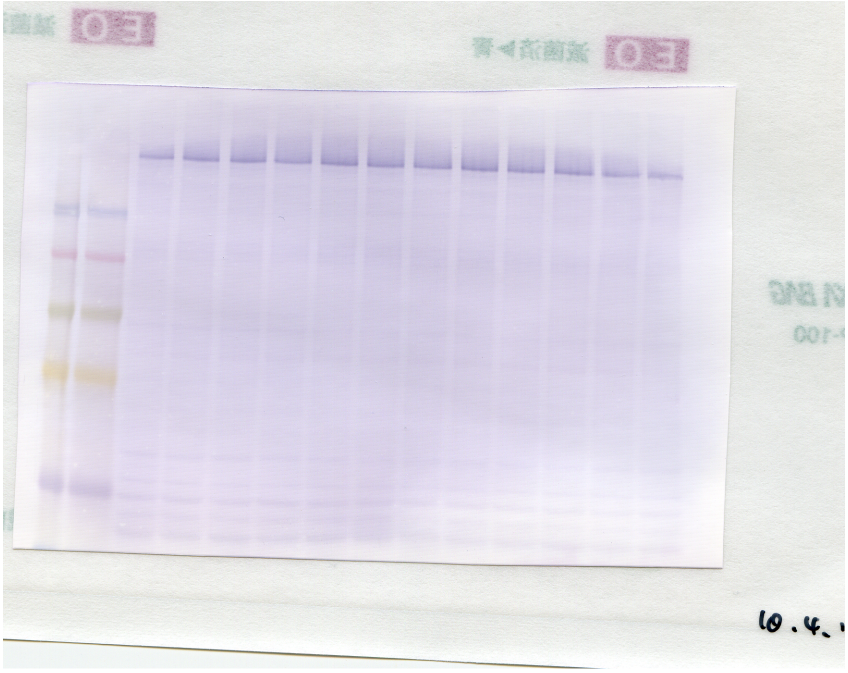

Fig. S2. BRCA1. uncropped

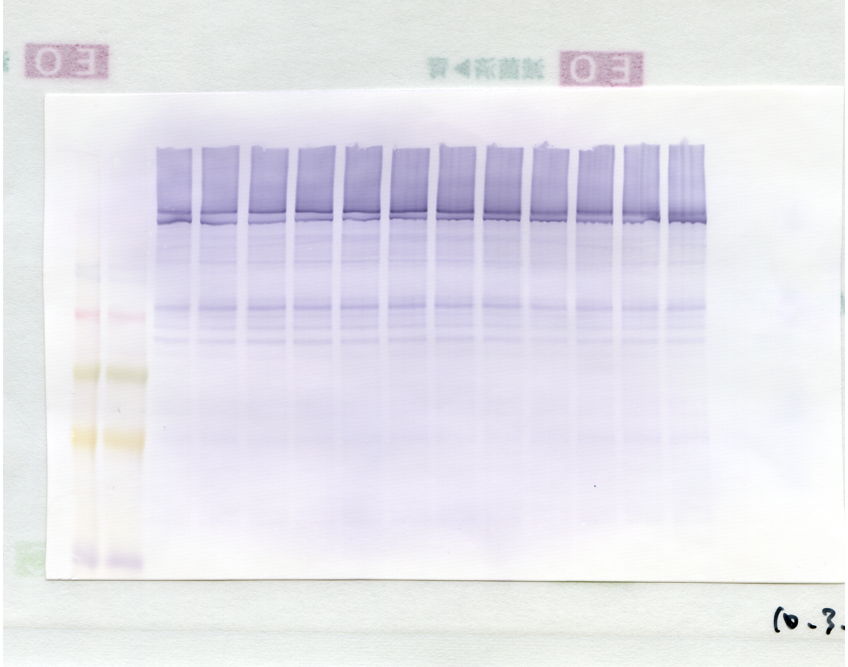

Fig. S2. BRCA2. uncropped

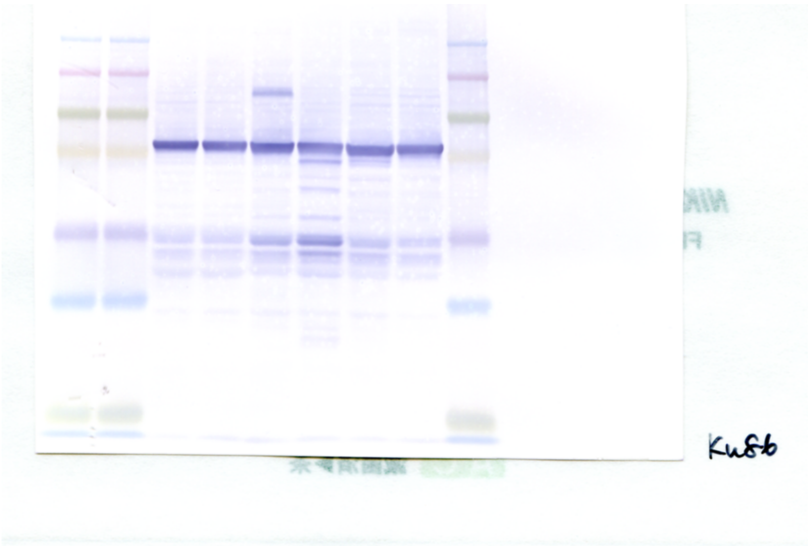

Fig. S2. Ku86. uncropped

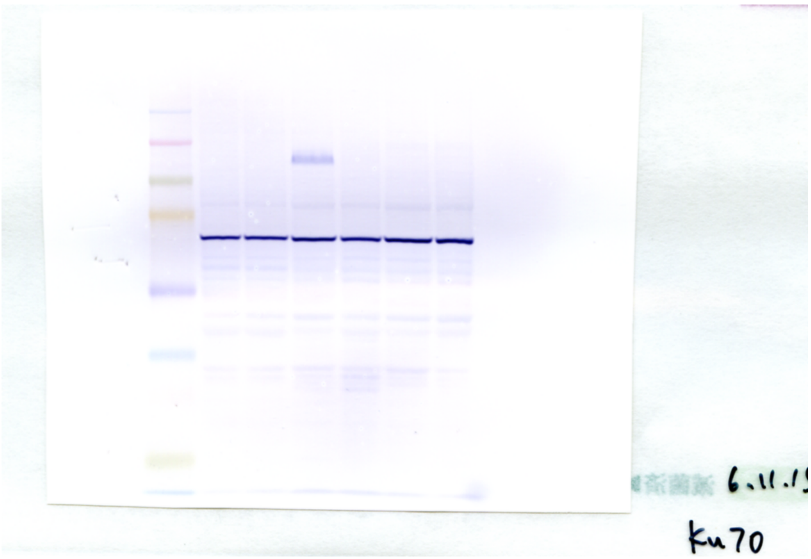

Fig. S2. Ku70. uncropped

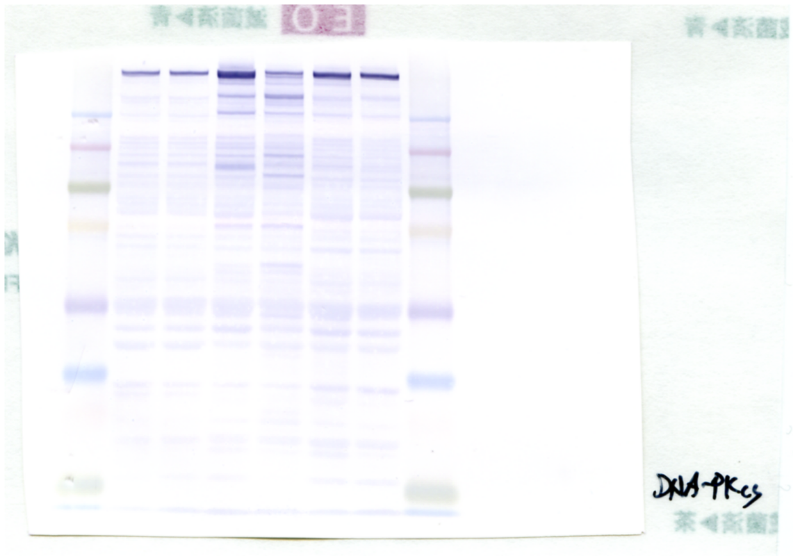

Fig. S2. DNA-PKcs. uncropped

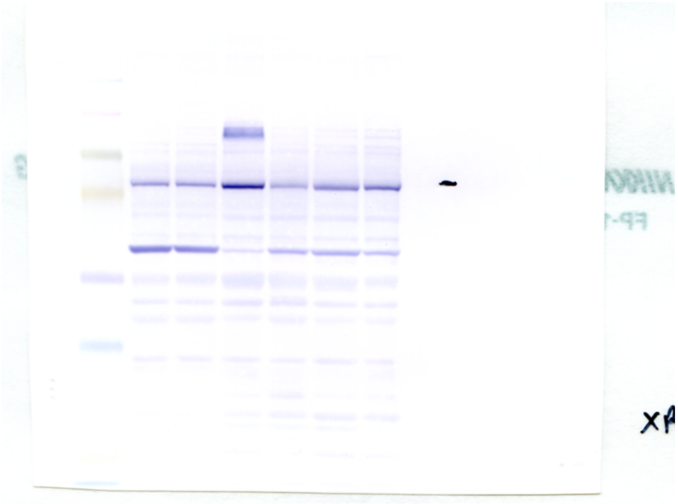

Fig. S2. XRCC4. uncropped

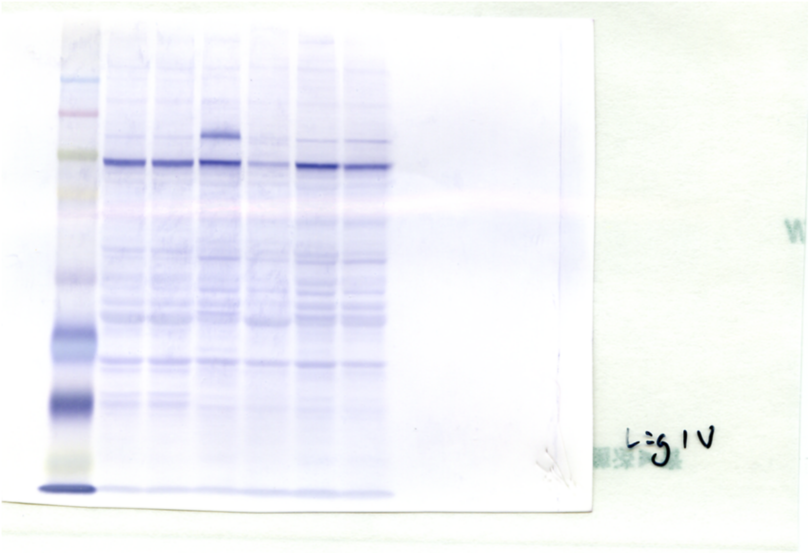

Fig. S2. LIG4. uncropped

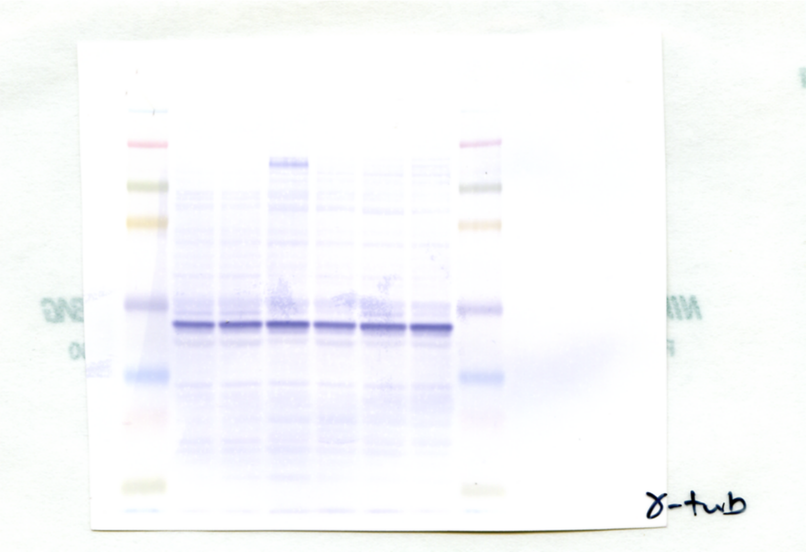

Fig. S2. γ-tubulin. uncropped
